# Risk Factors for Pancreatic Cancer in Patients with New-Onset Diabetes: A Systematic Review and Meta-Analysis

**DOI:** 10.3390/cancers14194684

**Published:** 2022-09-26

**Authors:** Claudia Mellenthin, Vasile Daniel Balaban, Ana Dugic, Stephane Cullati

**Affiliations:** 1 Department of Surgery, Hospital Fribourg, 1700 Fribourg, Switzerland; 2 Faculty of Science and Medicine, Institute of Family Medicine, University of Fribourg, 1700 Fribourg, Switzerland; 3 Arztpraxis am Bager, 3185 Schmitten, Switzerland; 4 Faculty of Medicine, Carol Davila University of Medicine and Pharmacy, 050474 Bucharest, Romania; 5 Department of Gastroenterology, Central Military Emergency University Hospital, 010825 Bucharest, Romania; 6 Department of Gastroenterology, Friedrich-Alexander-Universität Erlangen-Nürnberg (FAU), Medizincampus Oberfranken, 95445 Bayreuth, Germany; 7 Department of Medicine, Huddinge, Karolinska Institutet, 14186 Stockholm, Sweden; 8 Population Health Laboratory, University of Fribourg, 1700 Fribourg, Switzerland; 9 Department of Readaptation and Geriatrics, University of Geneva, 1211 Geneva, Switzerland

**Keywords:** pancreatic cancer, new onset diabetes, cancer screening, risk factors, meta-analysis

## Abstract

**Simple Summary:**

New onset diabetes patients are a high-risk group for pancreatic cancer. Since pancreatic cancer is responsible for less than 1% of new-onset diabetes cases, testing all of them might lead to an unfavorable risk/benefit balance. Additional risk factors can contribute to a better definition of the population that needs further screening. Currently, 22 studies examining additional risk factors have been published, but often they have a limited number of participants for the individual risk factor. By pooling their results in a meta-analysis, we could establish the magnitude of several risk factors. We found that pancreatic cancer cases were older than controls by 6.14 years (CI 3.64–8.65, 11 studies). Among new-onset diabetes patients, the highest risk of pancreatic cancer involved a family history of pancreatic cancer (3.78, CI 2.03–7.05, 4 studies), pancreatitis (5.66, CI 2.75–11.66, 9 studies), gallstones (2.5, CI 1.4–4.45, 4 studies), weight loss (2.49, CI 1.47–4.22, 4 studies), and high/rapidly increasing glycemia (2.33, CI 1.85–2.95, 4 studies) leading to more insulin use (4.91, CI 1.62–14.86, 5 studies). Risk factors or symptoms were distinct in the new-onset diabetes patient group. They are strongly connected to pancreatic cancer and are ideal for targeted screening, using a score or model as the first step.

**Abstract:**

(1) Background: Patients with new-onset diabetes (NOD) are at risk of pancreatic ductal adenocarcinoma (PDAC), but the most relevant additional risk factors and clinical characteristics are not well established. (2) Objectives: To compare the risk for PDAC in NOD patients to persons without diabetes. Identify risk factors of PDAC among NOD patients. (3) Methods: Medline, Embase, and Google Scholar were last searched in June 2022 for observational studies on NOD patients and assessing risk factors for developing PDAC. Data were extracted, and Meta-Analysis was performed. Pooled effect sizes with 95% confidence intervals (CI) were estimated with DerSimonian & Laird random effects models. (4) Findings: Twenty-two studies were included, and 576,210 patients with NOD contributed to the analysis, of which 3560 had PDAC. PDAC cases were older than controls by 6.14 years (CI 3.64–8.65, 11 studies). The highest risk of PDAC involved a family history of PDAC (3.78, CI 2.03–7.05, 4 studies), pancreatitis (5.66, CI 2.75–11.66, 9 studies), cholecystitis (2.5, CI 1.4–4.45, 4 studies), weight loss (2.49, CI 1.47–4.22, 4 studies), and high/rapidly increasing glycemia (2.33, CI 1.85–2.95, 4 studies) leading to more insulin use (4.91, CI 1.62–14.86, 5 studies). Smoking (ES 1.20, CI 1.03–1.41, 9 studies) and alcohol (ES 1.23, CI 1.09–1.38, 9 studies) have a smaller effect. (5) Conclusion: Important risk factors for PDAC among NOD patients are age, family history, and gallstones/pancreatitis. Symptoms are weight loss and rapid increase in glycemia. The identified risk factors could be used to develop a diagnostic model to screen NOD patients.

## 1. Introduction

The incidence of pancreatic ductal adenocarcinoma (PDAC) doubled over the last 2 decades [1]. The cumulative lifetime risk is 0.91% [2]. Diagnosis of PDAC comes too late for curative treatment in 80% of cases. This contributes to PDAC being one of the deadliest cancers worldwide, accounting for 4.7% of all cancer-related deaths [3]. Among diagnosed patients, the 5-year survival rate does not exceed 10% [4]. In countries that have screening programs for breast and colorectal cancers, PDAC has become the second most frequent cause of cancer mortality [5].

It has been established that all cancers discovered in the first years after diabetes diagnosis were already present and caused the diabetes, and several underlying mechanisms are under research [6,7,8,9,10,11,12]. Diabetes or prediabetes is often the first symptom of PDAC: diabetes diagnosis happens up to 3 years before the cancer diagnosis [13]. Among pancreatic cancer patients, about 80% have a diagnosis of either hyperglycemia or diabetes. Blood glucose levels slowly increase as early as 10 years before PDAC diagnosis, in the prediabetes range [14]. This has led to the idea that NOD or even prediabetes could be a potential clue to the early diagnosis of pancreatic cancer [15].

**Figure 1:**
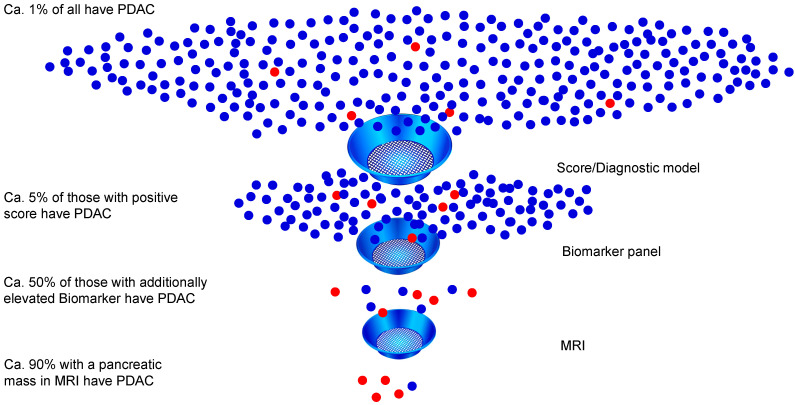
New onset diabetes patients—how to find those with pancreatic cancer.

As pancreatic cancer is responsible for less than 1% of NOD cases, using a biomarker test for every patient with NOD might lead to an unfavorable risk/benefit balance if the performance of the test is not exceptional [16] (Figure 1).

To further stratify the group that would need biomarker and then imaging testing, the use of a simple model or score is interesting. This strategy of 3 sieves would be more cost-effective and cause less harm than a strategy leaning on biomarkers and imaging alone.

Currently, 22 studies examining additional risk factors have been published, but often they have a limited number of participants for the individual risk factor. Pooling their results in a meta-analysis should increase the precision.

Based on a systematic review with meta-analysis, this paper aims to assess PDAC risk in NOD individuals and to identify risk factors among NOD patients, which are needed for a stepwise diagnostic strategy.

## 2. Materials and Methods

We performed a systematic literature search and last updated it in June 2022 in the three major databases, PubMed (RRID:SCR_004846), Embase (RRID:SCR_001650), and Google Scholar (RRID:SCR_008878), using the terms described in Appendix A. We did not apply any search restrictions. The study is registered in the inplasy study registry (INPLASY202220065).

We included observational studies (both cohorts and case-control studies) reporting on NOD patients and assessing additional factors regarding the risk of developing PDAC. Our objectives were to identify these risk factors that further enrich the NOD population in PDAC occurrence. Also, we aimed to analyze the risk of PDAC in NOD patients compared to non-diabetic persons.

We excluded studies with the sole focus on biomarkers or medication. We did not include case reports, small case series, reviews, opinions, or articles without an English abstract. When we found interesting conference abstracts, we searched with the author's names for follow-up publications, and, if relevant, included those. As the data was presented in a very heterogenous way, we sometimes contacted the authors for additional data to be included in their study. However, not all authors answered (Appendix A, Table A1 of studies excluded at the full-text screening).

Two team members voted blindly during each step of the paper selection and quality assessment and made consensus decisions, resolving conflicts by discussion.

**Figure 2:**
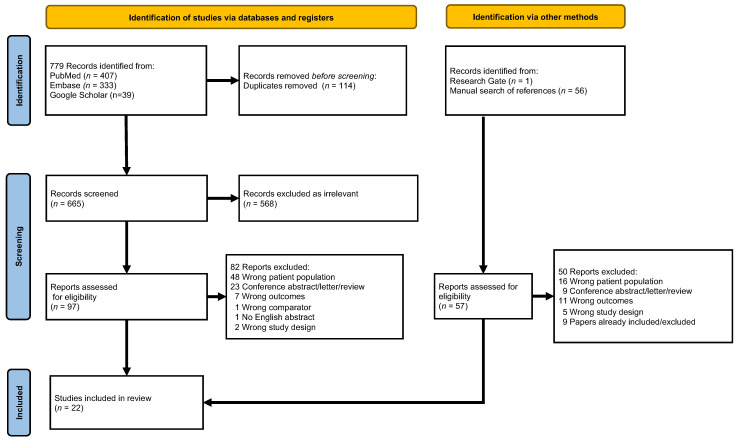
PRISMA flow diagram of the literature search and study selection process.

We extracted the following data from eligible studies: the name of the first author, journal and publication year, country and period, sample size, study type, patient characteristics, NOD definition, risk of PDAC in the NOD population, and additional risk factors (Figure 2).

### Data Analysis

For identifying studies and excluding duplicates, we used Covidence software (Veritas Health Innovation, Melbourne, Australia; RRID:SCR_016484), following the updated PRISMA 2020 guideline [17].

Studies reporting associations were used in the meta-analysis. Using the method of DerSimonian & Laird (an estimate of heterogeneity after the Mantel-Haentzel model), we performed a random-effects meta-analysis of risk factors that were reported in at least 3 papers either with a Risk Ratio or an Odds Ratio or with raw numbers that allowed us to calculate the Odds Ratio. All Confidence Intervals (CI) are 95%. All analyses were performed with STATA, version 16.1 (StataCorp, College Station, TX, USA).

**Figure A2:**
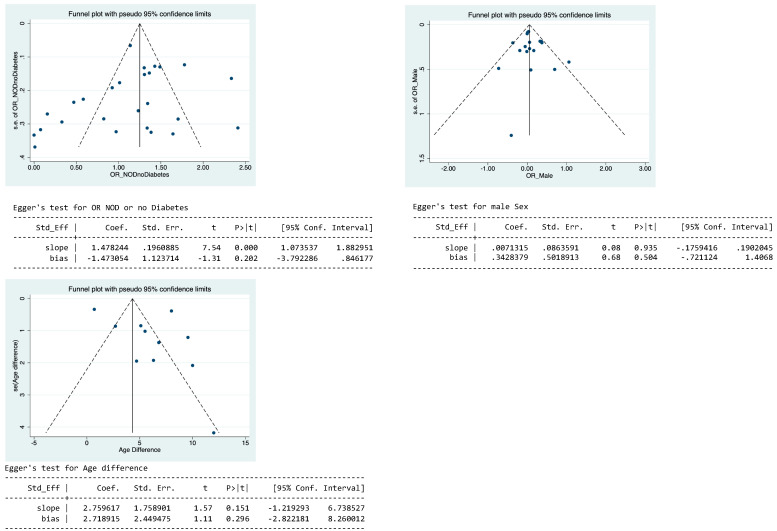
Funnel plots and Eggers test.

First, the authors (C.M. & V.D.B.) performed a quality assessment using 10 criteria as defined in the paper by Hoy et al. in a specific bias assessment tool for prevalence studies [18]. We judged overall bias for selected papers, following the corresponding bias flags among the 10 criteria. As the overall number of studies per risk factor was small, we did not exclude any study. To determine the risk of publication bias, we used a funnel plot and the Egger test (Appendix A, Figure A2).

We extracted data on the definition of NOD/subgroups of duration, age, sex, ethnicity, lifelong smoking, alcohol abuse, family history of PDAC, gall stones/cholecystitis, pancreatitis, a rapid increase of glycemia, weight loss, insulin use, obesity, and hyperlipidemia. When more than 2 groups were reported, we combined groups, for example, former smokers + current smokers = lifelong smokers. Or introduced the most meaningful cut-off; for example, for groups of BMI (Body Mass Index) reported, we distinguished BMI < 30 = not obese, BMI ≥ 30 obese (Details in Appendix A).

We also extracted the percentage of NOD patients that developed PDAC (in the cohort studies) and the OR for PDAC for NOD versus no diabetes in the case-control studies.

## 3. Results

### 3.1. Studies

The search yielded 779 references, which we imported into Covidence. After removing duplicates and excluding irrelevant studies, we selected 15 studies for data extraction. Reference lists and citation searches (for studies that cited those we had already included) provided an additional 6 studies to be included in the analysis. There was one paper from other sources. Twenty-two studies were included. In total, 576,210 patients with NOD contributed to the analysis, of which 3560 had PDAC (Figure 2).

The study designs were heterogeneous, including retrospective cohorts (some with prospective analysis) (*n* = 13), case-control studies (*n* = 8), and one small prospectively recruited screening study, with recruitment at a diabetes clinic [19] (Table 1).

### 3.2. Risk Factors for PDAC in NOD Patients

**Figure 3:**
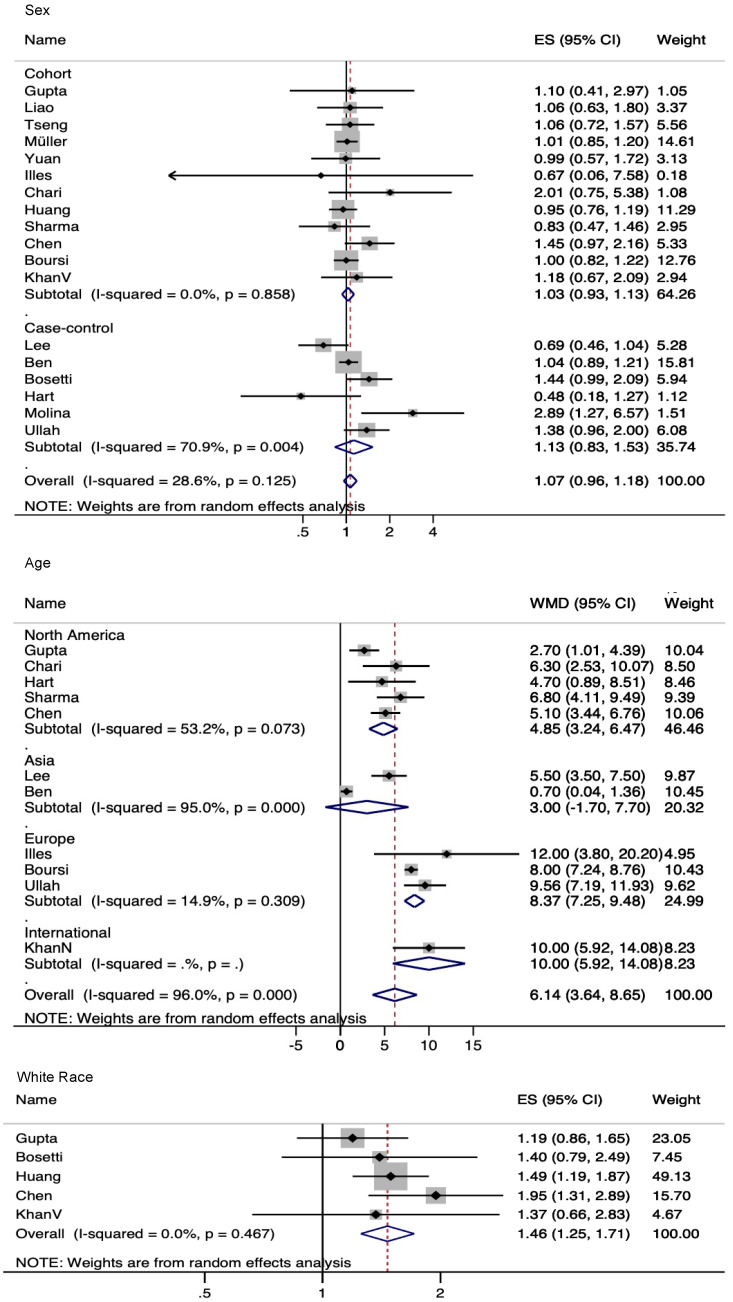
Meta-analysis of demographic risk factors.

The strongest demographic risk factor was older age. The overall mean age difference in the studies was more than 6 years (pooled age mean difference 6.14 years, CI 3.64–8.65, I^2^ = 96%, 11 studies), which seemed to be even more pronounced in European studies. Sex was not a statistically significant risk factor: the overall effect size (ES, either from odds ratio or incidence rate ratio) in overall studies was 1.07 for the male gender (CI 0.96–1.18, I^2^ = 28.6%, 18 studies). Race was analyzed in only a few studies, which showed that whites had a slightly higher risk for PDAC (ES 1.46, CI 1.25–1.71, I^2^ = 0.0%, 5 studies) (Figure 3).

Concerning other risk factors, smoking was a just barely statistically significant risk factor (ES 1.20, CI 1.03–1.41, I^2^ = 44.0%, 9 studies); the same was true for alcohol (ES 1.23, CI 1.09–1.38, I^2^ = 5.9%, 9 studies). Pancreatitis (ES 5.66, CI 2.75–11.66, I^2^ = 85.5%, 9 studies) and gall stones/cholecystitis (ES 2.5, CI 1.4–4.45, I^2^ = 87.0%, 4 studies) showed an increased risk. Positive family history of pancreatic cancer was a very strong risk factor, with an effect size of 3.78 (CI 2.03–7.05, I^2^ = 68.6%, 4 studies). Obesity (defined as BMI ≥ 30) was not associated with more pancreatic cancer cases within the studied populations of NOD (ES 0.67, CI 0.45–1, I^2^ = 84.3%, 5 studies).

**Figure 4:**
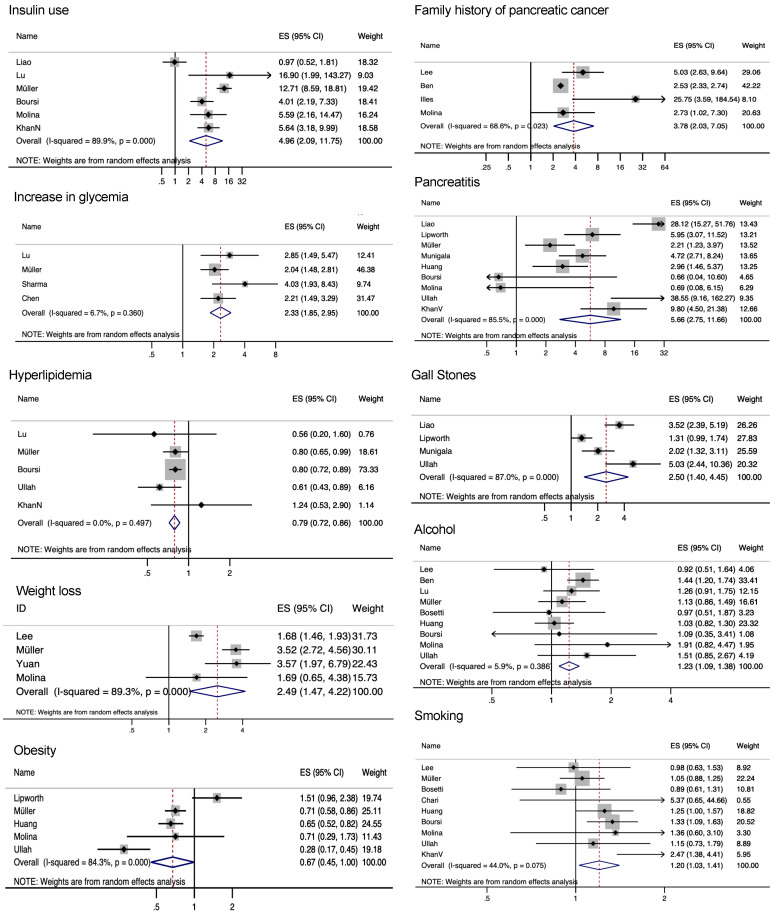
Meta-analysis of other risk factors/symptoms.

Weight loss was a significant symptom, with an effect size of 4 (CI 3.1–4.9, I^2^ = 89.3%, 4 studies). A rapid increase in glycemia was significant in 7 studies [22,27,28,35,36,41,42], but it was reported with such heterogeneity that a meta-analysis was impossible for all studies (ES 2.33, CI 1.85–2.95, I^2^ = 6.7%, 4 studies). The rapid increase in glycemia could lead to more insulin use (ES 4.91 CI 1.62–14.86 I^2^ = 91.9%, 5 studies) in pancreatic cancer patients. A few studies showed a negative association with high blood lipids [19,25,26,38,42] (Figure 4).

### 3.3. Association between NOD and PDAC

All studies identified a strong association between NOD and PDAC. The overall effect size was 3.35 (CI 2.75–4.09, I^2^ = 83.3%), with a clear tendency of the ES to be higher when the interval since NOD diagnosis was shorter: in the first year after diabetes diagnosis, it was 5.52 (CI 3.61–8.46, I^2^ = 85.6%).

### 3.4. Proportion of NOD Caused by PDAC

**Figure 5:**
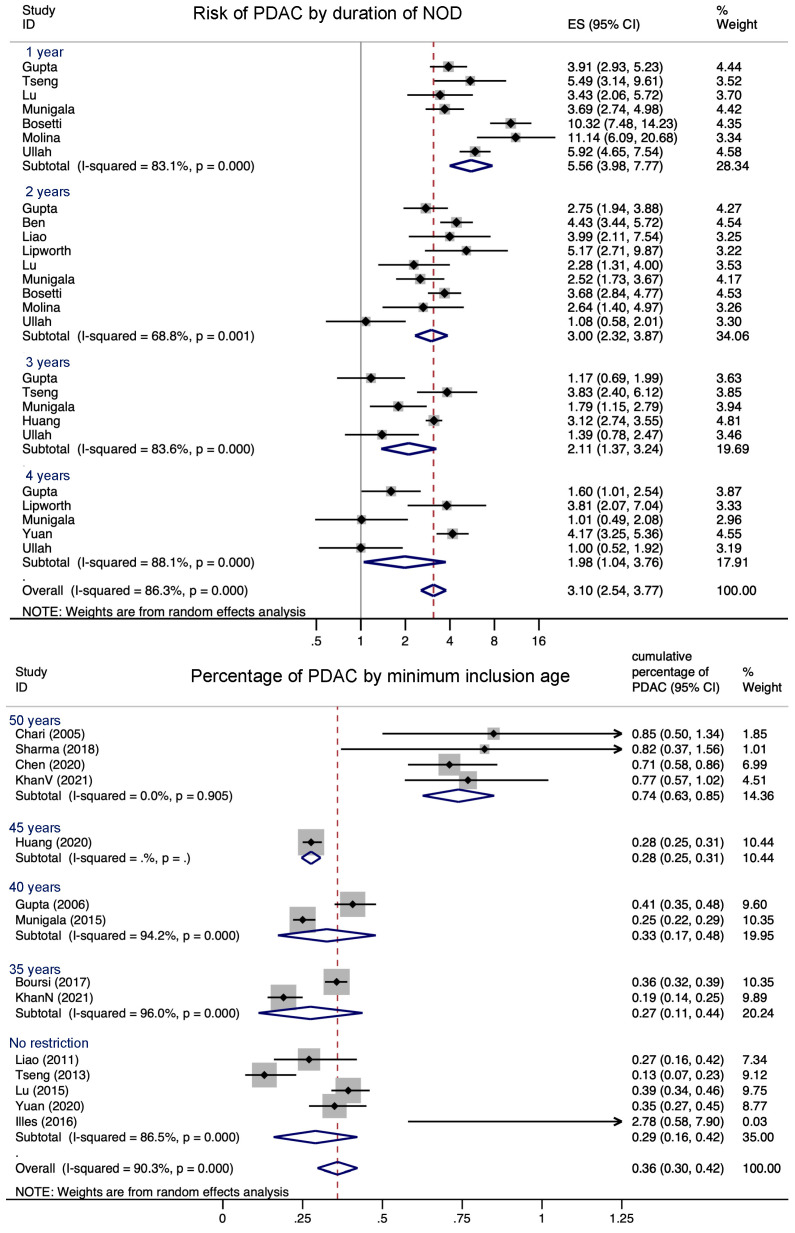
Meta analysis of OR of PDAC in NOD as opposed to no diabetes in patients grouped by the allowed duration of NOD as defined in the study or by the corresponding subgroup. The proportion of NOD with pancreatic adenocarcinoma as a probable reason for diabetes in the cohort studies in subgroups of applied age restriction. When only NOD older than 50 were included, it was highest.

As we assumed that all PDAC was present before the diabetes diagnosis and the cause of NOD, we calculated the cumulative percentage of observed PDAC diagnosis. It ranged from 0.13% in the Taiwanese registry study by Tseng et al. [25] to 2.7% in the prospectively recruited screening study by Illés et al. [19]. Studies excluding people under 50 found 0.74% (CI 0.63–0.85%) of PDAC cases among NOD patients. The overall cumulative percentage of PDAC in NOD patients was 0.36% (CI 0.3–0.42, I^2^ = 86.3%, 14 studies) (Figure 5).

## 4. Discussion

### Limitations and Strengths of the Study

Despite our systematic approach, we could have overlooked critical studies through our choice of search terms. We minimized this by using several formulations and searching references regarding the included papers. The most significant limitations of our findings are biases in the included studies and the disparity of representation of geographical regions. Many studies are from the USA, Europe, and Asia, and one is from Australia, but we could not identify any South American or African studies.

In extracting the data, we were limited by the heterogeneity of the included studies. To have enough data to analyze, we included studies with slightly different definitions. Definitions of how we extracted the data are in Appendix A. The results of our meta-analyses still show considerate heterogeneity, partially explained by the difference in inclusion criteria (for example, age), ethnicity, and definition of new-onset diabetes, all of which we also examined as risk factors or subgroups. Some risk factors might also interact with each other.

A strength of our review is that it gives a complete, systematic overview of the current body of evidence regarding additional risk factors for PDAC in NOD populations. Our paper is, to our knowledge, also the first to conduct a meta-analysis on the risk factors.

## 5. Conclusions

### 5.1. Interpretation of Findings

The association between diabetes and PDAC has long been recognized. Several papers have shown that the risk is highest directly after diagnosis and then decreases over subsequent years [41]. The association might be confounded by commonly shared risk factors such as obesity or chronic pancreatitis. The actual frequency of pancreatic cancer in the population of NOD is still unclear, as most studies are retrospective, and the percentage in the only prospective study is much higher. Currently, four prospective studies are recruiting patients and might bring more clarity [42,43,44,45].

It is essential to look specifically at the group of NOD patients, as they differ from the general population. For instance, NOD patients tend to be more obese than the general population, as obesity is a very important risk factor for diabetes mellitus. Within the population of NOD patients, obesity is not associated with more PDAC cases, as our analysis shows. In fact, the mean BMI of pancreatic cancer cases was lower than that of NOD controls. This might be even more pronounced through tumor-induced recent weight loss. It was surprising to find at most a weak association of smoking and alcohol abuse in this meta-analysis. Possibly these risk factors are more important for non-diabetic PDAC patients, or their importance has generally been overestimated.

Risk factors or symptoms that are distinct in the NOD patient group and are strongly connected to pancreatic cancer are ideal for targeted testing. They can be used for statistical model fitting. Our analysis showed that age, family history of PDAC, pancreatitis/cholecystitis, weight loss, and rapid increase in glycemia/necessity of insulin are robust candidates. A tendency to lower lipids, unusual in newly diagnosed diabetes patients, is also interesting. Unfortunately, some of the strongest risk factors are rather rare, which negatively impacts the sensitivity of such models. The correct balance between the frequency and magnitude of those risk factors remains to be found.

### 5.2. Importance of the Presented Work and Future Directions for Early Diagnosis Programs

Screening programs aim to diagnose cancer in the asymptomatic, early stages amenable to curative treatment. Scrutiny regarding balancing benefits and burdens, cost, survival extension, and quality-life years gain is essential. As pancreatic cancer has a low incidence in the total population, this is a challenge. The main risk of pancreatic cancer screening is a too-high rate of false-positive results, leading to unnecessary further investigations. Including the identified additional risk factors or symptoms can help define the target population.

A stepwise approach of first identifying a group with increased risk of pancreatic cancer within the NOD population through a scoring or diagnostic model and then further reducing the number of patients needing imaging by a biomarker test has been proposed by Pannala et al. [15]. Several studies have proposed scores to identify the best group for testing [19,22,35,36]. A scoring system has advantages, as it is objective and can be validated. Nevertheless, it also has disadvantages, such as being time-consuming for the family physician or challenging to apply when data is missing. The complexity of a scoring model should consider the balance between the accuracy of prediction and the simplicity of daily use. Considering the slightly different associations of risk factors in different regions (for example, the USA, Europe, Asia), such scoring might differ depending on the location. These regional differences are related not only to the characteristics of PDAC patients but also to NOD. Diabetes is closely related to diet and obesity, which are subject to socio-cultural and genetic influences. In the USA, the average age for diabetes diagnosis is lower than it is in Europe. In Asia, patients with a much lower BMI than that in western countries suffer from an increased risk for diabetes [46]. In conclusion, before using a score as a diagnostic model in a new population, it will need adaptation, or at least calibration and validation.

## Figures and Tables

**Table 1:** Studies, designs, and populations (References in brackets).

Author, Journal, Year	City or Region, Country, Database Name (When Available), Period (Years)	Study Design, Study Population, Sampling Method	Patient Characteristics in NOD (Mean Age, Obesity, Smoking)	NOD Definition
Gupta et al., Clin Gastroenterol Hepatol, 2006 [20]	USA, VA National Patient Care database 1998–2004	Retrospective cohort, veterans, all without previous diagnosis of PDAC or DM were included, 36,631 developed NOD, of which 149 had PDAC	US veterans > 40 years, in NOD cohort 97% male, average age 64 years	1 year
Boursi et al., Gastroenterology, 2017 [21]	UK, THIN database 1995–2013	Retrospective cohort, all patients with incident DM were included: 109,385 patients with NOD, of which 390 had PDAC	All < 35 years were excluded	<3 years
Lee et al., Journal of Clinical Gastroenterology 2012 [22]	Seoul, Korea, 2003–2009	Retrospective case-control Cases: 151 NOD with PDAC, Controls: 302 NOD, no cancer 1:2 matched, randomly selected	Mean age 61 years (cases) and 56 years (controls) 58% male in cases, 66% in controls	<2 years
Ben et al., European Journal of Cancer 2011 [23]	Shanghai, China, Hospital Data 2000–2009	prospective case-control Cases: 1458 PDAC, of which 307 NOD Controls: 1:1 matched for time of admission, age, sex, sociodemographic variables, 1528 of which 88 NOD	Mean age 62 years 67% male	<2 years
Liao et al., Journal of Gastroenterology and Hepatology 2012 [24]	Taiwan, National Health Insurance 1998–2007	Retrospective cohort, entire population, nested case-control: Cases: all DM, of which 6911 had NOD, and 19 PDAC Controls: No DM, 1:4 matched for age and sex, randomly selected	Mean age of 55.9 years 54% male, Obesity 2.43%	<2 years
Tseng et al., Pancreas 2013 [25]	Taiwan, National Health Insurance 2005–2006	Retrospective cohort, general population, random sample including 29,236 NOD and of those 32 PDAC	48.5% male	Groups 1, 3 or >3 years
Lipworth et al., Diabetes/Metabolism Research and Reviews 2011 [26]	Milan, Italy 1983–1992; 1991–2008	Combined data from two prospective case-control studies, hospital population, convenience sample, including 51 PDAC/NOD cases and 39 NOD controls	Median age 55 years (controls), 63 years (cases) 63% resp. 53% male	Subgroup < 2 years
Lu et al., British Journal of Cancer 2015 [27]	UK, THIN Database 1996–2010	Two retrospective cohorts from the general population NOD cohort 44,373, of which 175 had PDAC Control-cohort: 188,734 had no diabetes, of which 354 had PDAC	Mean age ~70 years (age groups) 58% male, 35% obesity, 23% current smokers	Groups 1, 2, 5, and >5 years
Müller et al., Pancreatology 2019 [28]	Great Britain, Clinical Practice Research Datalink (CPRD) 2004–2013	retrospective case-control Cases: 588 PDAC and NOD Controls: 5486 NOD, 1:10, matched for age, sex, time DM diagnosis, follow up	Mean age ~70 years (age groups), 49.5% male, 28.7% BMI > 30, 18% current smokers	<2 years
Munigala et al., Clinical and Translational Gastroenterology 2015 [29]	St Louis, USA, Veterans’ Health Administration national medical care data sets 1998–2007	Retrospective cohort, veterans, all without previous diagnosis of PDAC or DM, were included. 73,811 developed NOD, of which 183 had PDAC	Mean age 60.2 years, all < 40 years excluded by design 94% male, 74% white 46.8% obesity, 57% smoking	Groups 1, 2, 3, 4 years
Yuan et al., JAMA Oncology, 2020 [30]	USA Nurses’ Health Study (NHS), baseline 1978, Health Professionals Follow-Up Study (HPFS), baseline 1988	Two retrospective cohorts, female nurses and male physicians, without previous diagnosis of PDAC or DM. Within the patients with NOD, 67 PDAC cases were observed.	Mean age 69 years White 93.3%, Black 3.5% Obesity 43% Ever-smokers 56%	<4 years
Bosetti et al., Annals of Oncology 2014 [31]	International, USA, Canada, Greece, Central Europe, Italy, Australia, 1983–2012	Combined data from 15 case-control studies Cases: PDAC Controls: hospital/hospital visitors/populationNOD subgroup; including 525 NOD/PDAC cases	Not published for NOD subgroup	Groups < 1 years, 1–2, 2–5, >5
Illés et al., Pancreatology, 2016 [19]	Szeged, Hungary 2012–2014	Prospectively recruited, 108 patients with NOD, of which 3 had PDAC	Mean age 58 years 42.6% male, mean BMI 30.5, 29% ever smoker	<3 years
Chari et al., Gastroenterology 2005 [32]	Rochester, USA 1950–1994	Cohort of 2122 NOD including 18 PDAC with nested case-control: Cases: NOD with PDAC, 18 cases Controls: NOD, 1:4 matched for age, sex, time of diabetes diagnosis, 72 controls	All < 50 years excluded by design No demographic data on cohort	<3 years
Hart et al., Pancreas 2011 [33]	Rochester, USA 1981–2007	Retrospective case-control, 29 Cases: all NOD and PDAC in a 120-mile radius of Rochester 43 Controls: NOD matched for sex and age	Mean age 76 years cases, 72 years controls, 37% male cases, 56% controls	<3 years
Huang et al., Clinical Gastroenterology and Hepatology, 2020 [34]	Kaiser Permanente Southern California, USA (KPSC, Insurance) 2006–2016	Retrospective cohorts, all with sufficient data and without previous diagnosis of PDAC, were included. 110,699 NOD, of which 306 with PDAC	All < 45 years were excludedMean age 59 years, Male 52% Whites (44%), Hispanics (37%), Asians (15%) Blacks (15%).	<3 years
Sharma et al., Gastroenterology 2018 [35]	Rochester, USA, Rochester Epidemiology Project (REP) 2000–2015	Retrospectively collected data from 4 independent cohorts, with 64 PDAC/NOD and 192 NOD-Controls in the discovery set, and a cohort of 1096 NOD, including 9 PDAC in the validation set	All < 50 years were excluded Mean age 65.6 years, 50% male	<3 years
Chen et al., Digestive Diseases and Sciences 2021 [36]	Kaiser Permanente Southern California, USA (KPSC, Insurance) 2010–2014	Retrospective cohort of all patients without previous diagnosis of PDAC, meeting NOD criteria during the enrolment period, 13,947 NOD including 99 PDAC	All < 50 years were excluded No PDAC: 64.1 years, 48% male, 91 kg, PDAC: 69.2 years, 57% male, 84.4 kg	<3 years
Molina-Montes et al., Gut, 2021 [37]	PanGenEU, Europe, 28 centers from Spain, Italy, UK, Ireland, Germany, Sweden 2007–2014	Retrospective case-control, we used only data from the subgroup with NOD, with general population as control. Data on long-standing diabetes was ignored. It included 200 cases of PDAC/NOD	63.4% male, mean age ~65 years (age groups), 30.5% obese	<2 years
Khan, Pancreatology, 2021 [38]	TrinetX—Validation of ENDPAC	Retrospective cohort of 15,539 NOD patients, of which 48 had PDAC	<50 years excluded by design PDAC 68 years, 54% male, 81% white, 39% smokers No PDAC, 67 years, 50% male, 76% white, 21% smokers	<3 years
Khan, Pancreas, 2021 [39]	TrinetX—validation of Boursi	Retrospective cohort of 27,893 NOD patients, of which 52 had PDAC	<35 years excluded by design PDAC 74 years, No PDAC 64 years	<3 years
Ullah, BMC Cancer, 2021 [40]	EL-PaC-Epidem London, UK 2008–2020	Case-Control study, 965 PDAC, 3963 Non-malignant pancreatic disease, 4355 Controls	Mean age 55.1, 51% male, 54.4% white	Groups 1,2,3 years

**Figure A1:**
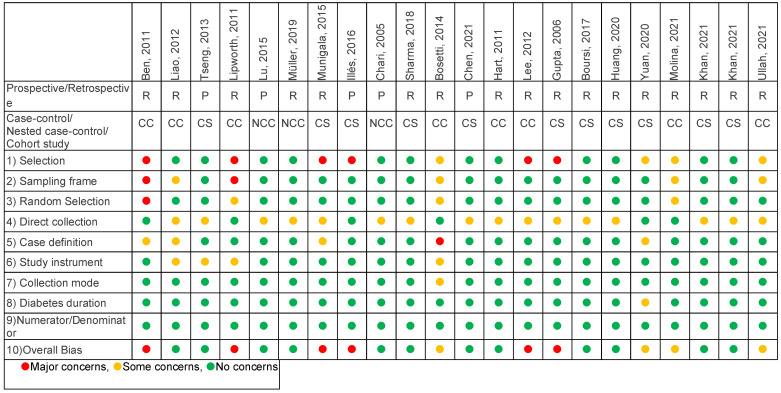
Bias assessment of publications. Risk of Bias Assessment.

## Data Availability

For access to data, contact the corresponding author.

## Appendix A

### Appendix A.1. Search Terms

Pubmed search of:

(((“carcinoma, pancreatic ductal”[MeSH Terms] OR (“pancreatic neoplasms”[MeSH Terms] OR (“pancreatic”[All Fields] AND “neoplasms”[All Fields]) OR “pancreatic neoplasms”[All Fields] OR (“pancreatic”[All Fields] AND “cancer”[All Fields]) OR “pancreatic cancer”[All Fields])) AND (“probability”[MeSH Terms] OR “risk factors”[MeSH Terms] OR “diagnostic techniques and procedures”[MeSH Terms]) AND “diabetes mellitus, type 2”[MeSH Terms]) AND hasabstract[text]).

Embase search of:

Pancreas carcinoma AND non-insulin-dependent diabetes mellitus AND high-risk population

OR

Pancreas carcinoma AND non-insulin-dependent diabetes mellitus AND risk assessment.

Google scholar search of:

All in the title: diabetes risk OR diagnosis “pancreatic cancer”.

After the selection of relevant articles, we also checked their references for additional possible matches with our research topic, which had been missed in the initial search, and checked for publications that cite those we previously included. Additional papers that were already known to the authors or came to their knowledge from other sources were also included.

### Appendix A.2. Effect Size of PDAC in NOD Patients versus No Diabetes Patients

Each study had a different definition of NOD. Some used a definition of 1 year, others 2, 3, or 4 years after diabetes diagnosis (Table 1). Other studies had several subgroups for the duration of diabetes. This variability of definitions influenced the results considerably. For that reason, we did a subgroup analysis, either with the subgroups as published or with the definition used in the study. This has the disadvantage that a study without subgroups and using a 3-year definition will have in that group patients with diabetes onset less than a year ago—that is not reported separately so we cannot know that.

### Appendix A.3. Parameters for Meta-Analysis, Remarks about the Reported Risk Factors/Symptoms

There was considerable heterogeneity between the studies and the published values. To include as many different studies as possible, we did a meta-analysis of the reported effect size. Where a crude Odds Ratio was reported, we took this. When possible, we calculated an Odds Ratio from the published case frequency numbers. In a small number of studies, only a Relative Risk or Incidence Ratio was published, and there were no case numbers to calculate an Odds Ratio. In this case, we used the published Effect size.

Smoking

3 studies of the 6 with data on smoking reported 3 categories: never smokers, ex-smokers, and current smokers; the others only 2 categories, exposed or not exposed. We put all patients that were ever exposed to smoking into one group.

Alcohol abuse

The reporting on alcohol consumption was also heterogenous, with 2, 3, or 4 different exposure groups. To group participants according to their alcohol status, we used the cut-off of 20 g/day of risky consumption (independent of gender) and sorted the published groups accordingly.

Obesity

Whenever several groups of body mass index were reported, we introduced dichotomous sorting with the limit of body mass index equal to or above 30 as the definition of “obesity”.

Pancreatitis

Some studies reported on “chronic pancreatitis” and others on status “post pancreatitis”, but as there were few studies, and acute pancreatitis can lead to chronic pancreatitis, we analyzed them together.

Gall stones/Cholecystitis

Some studies reported on Gall stones, others on Cholecystitis. We grouped those together under “Gall stones”, as Cholecystitis without Gall stones is very rare.

Rapid increase/High Glycemia

Here the heterogeneity was huge, as some papers reported means and differences in means, others a slope, and third a proportion. Some referred to HbA1c, others to fasting glucose. To be able to meta-analyze it at all, we used all papers that reported numbers of patients, though some reported the numbers with a rapid increase [27,28], while others reported those with high fasting glucose (>160 mg/dL) at diagnosis [35,36].

Insulin use

Medication was not the focus of our review, and studies that looked solely at medication were excluded, so we have not included all studies that look at the association of insulin use.

### Appendix A.4. Bias Assessment

External validity

The most relevant concern was selection bias. Some studies [19,23,26] sampled selectively from hospital populations that were probably not representative of the general population. Other studies [27,29] examined military veterans, a rather specific cohort comprising predominantly males and not representative of the overall population.

The choice of controls was also prone to some bias, as in some studies [26,28], convenience samples were used. The controls in Ben et al. [23] consisted of a hospital population. Moreover, they excluded all malignant diseases and all patients with diagnoses related to alcohol, tobacco, and drugs, which introduces considerable bias in assessing risk factors.

Internal validity

The registry studies, which did not collect data directly from the patients, are at risk of misclassifications. Generally, the retrospective assessment of records is problematic because missed diagnoses regarding PDAC and diabetes might lead a study to underestimate the connection between those two diseases (Figure A1).

External validity

Was the study’s target population a close representation of the national population in relation to relevant variables?

Was the sampling frame a true or close representation of the target population?

Was some form of random selection used to select the sample/the control, or was a census undertaken?

Internal validity

Were data collected directly from the subjects (as opposed to a proxy)?

Was an acceptable case definition used in the study?

Was the study instrument that measured the parameter of interest shown to have validity and reliability?

Was the same mode of data collection used for all subjects?

Was the length of the longest duration for the parameter of interest (NOD)appropriate?

Were the numerator(s) and denominator(s) for the parameter of interest appropriate?

Overall risk of bias

For assessing the overall risk of bias, we considered patient selection as the most crucial factor, which dominated our decision.

### Appendix A.5. Publication Bias

To test for publication bias (done only for effects reported in at least 10 studies), we calculated a funnel plot for the effect size of PDAC in the NOD population (11 studies with 26 OR (different age groups) reported this), the age difference (reported by 10 studies), and for the effect size of sex as a risk factor within the NOD subgroup (reported by 18 studies). There was no suspicion of relevant publication bias (Figure A2).

**Table A1:** List of studies excluded at the full-text screening stage, with brief reasons.

	First Author, Year	Journal	Title	Reason for Exclusion	Notes
1	Ballotari, 2017	BMC Cancer	Diabetes and risk of cancer incidence: Results from a population-based cohort study in northern Italy.	Wrong population	
2	Zhang, 2018	Diabetes/Metabolism Research and Reviews	Clinical features and risk factors for cancer in patients with type 2 diabetes in Qingdao, China.	Conference abstract/letter/review	
3	Arthur, 2019	Annals of Epidemiology	Adiposity, history of diabetes, and risk of pancreatic cancer in postmenopausal women.	Wrong population	
4	Gullo, 1999	Annals of Oncology	Diabetes and the risk of pancreatic cancer.	Wrong outcomes	No other risk factors are looked at.
5	Jamal, 2009	World J of Gastroenterology	Diabetes mellitus as a risk factor for gastrointestinal cancer among American veterans.	Wrong population	
6	Gul, 2010	The American J of the Medical Sciences	Ca 19-9 levels in type 2 diabetes mellitus patients.	Wrong outcomes	Not about pancreatic cancer. Conference abstract, uploaded the corresponding article.
7	Brodovicz, 2011	Pharmacoepidemiology and Drug Safety	Synergistic effect of type 2 diabetes (T2D) and history of pancreatitis on pancreatic cancer risk: A retrospective cohort study from the general practice research database (GPRD).	Conference abstract	Abstract, unable to find paper
8	Hense, 2011	Diabetology and Metabolic Syndrome	Cancer incidence in type 2 diabetes patients—First results from a feasibility study of the D2C cohort.	Wrong population	Could not find a sub-analysis on PDAC, only overall risk for cancer according to diabetes duration
9	Gong, 2012	World J of Gastroenterology	ABO blood type, diabetes, and risk of gastrointestinal cancer in Northern China.	Wrong patient population	
10	Henry, 2012	Cancer Research	History of diabetes mellitus as a risk factor for pancreatic cancer: The Iowa Women's Health study.	Conference abstract	Paper that followed had wrong patient population
11	Andersen, 2012	Diabetes/Metabolism Research and Reviews	The practical importance of recognizing pancreatogenic or type 3c diabetes.	letter	Letter about an article, imported it into full-text review.
12	Honjo, 2012	Epidemiology/Genetics	An observational prospective study of cancer in Japanese subjects with type 2 diabetes with special reference to pancreatic cancer.	Conference abstract	Abstract, unable to find paper
13	Elena, 2013	Cancer Causes and Control	Diabetes and risk of pancreatic cancer: A pooled analysis from the pancreatic cancer cohort consortium.	Wrong population	
14	Suceveanu, 2015	Pancreatology	Diabetes mellitus, obesity, and chronic pancreatitis? Independent risk factors for pancreatic adenocarcinoma (PAC) in the Romanian Black Sea coast area.	Conference abstract	Abstract, unable to find paper
15	Mansoor, 2016	Gastroenterology	Risk factors for pancreatic cancer in new-onset diabetes mellitus: A population-based study.	Conference abstract	Abstract, unable to find paper
16	DeJong, 2016	Gastroenterology	Gastrointestinal cancer incidence in type 2 diabetes mellitus; results from a large retrospective population-based cohort study.	Wrong population	
17	Lu, 2016	British J of Cancer	Reply to Comment on "New-onset type 2 diabetes, elevated HbA1c, antidiabetic medications, and risk of pancreatic cancer".	letter	Answer to a comment over the paper
18	Dugnani, 2016	Pancreatology	Diabetes associated with pancreatic ductal adenocarcinoma is just diabetes: Results of a prospective observational study in surgical patients.	Wrong population	
19	Attner, 2012	Cancer Causes & Control	Cancer among patients with diabetes, obesity and abnormal blood lipids: a population-based register study in Sweden.	Wrong population	The population are cancer cases, not NOD, and diabetes is the outcome.
20	Mizuno, 2013	J of Gastroenterology	Risk factors and early signs of pancreatic cancer in diabetes: screening strategy based on diabetes onset age.	Wrong patient population	
21	Zhang, 2012	BMC Public health	Increased risk of cancer in patients with type 2 diabetes mellitus: a retrospective cohort study in China.	Wrong population	
22	Magliano, 2012	European J of Endocrinology	Incidence and predictors of all-cause and site-specific cancer in type 2 diabetes: the Fremantle Diabetes Study.	Wrong population	
23	Lai, 2013	The J of Clinical Endocrinology and Metabolism	The association between self-reported diabetes and cancer incidence in the NIH-AARP Diet and Health Study.	Wrong population	
24	Tseng, 2013	Acta Diabetologica	Diabetes, insulin use, smoking, and pancreatic cancer mortality in Taiwan.	Wrong population	
25	Ahn, 2013	The Korean J of Gastroenterology	[New-onset diabetes as an early sign of pancreatic cancer].	review	Article in Korean google translate shows that it is a review.
26	Valent, 2015	J of Diabetes and Its Complications	Diabetes mellitus and cancer of the digestive organs: an Italian population-based cohort study.	Wrong population	
27	Kolb, 2009	Cancer Biology & Therapy	Glucagon/insulin ratio as a potential biomarker for pancreatic cancer in patients with new-onset diabetes mellitus.	Wrong population	
28	Ogunleye, 2009	British J of Cancer	A cohort study of the risk of cancer associated with type 2 diabetes.	Wrong population	
29	Hemminki, 2010	The Oncologist	Risk of cancer following hospitalization for type 2 diabetes.	Wrong population	no NOD
30	Ben, 2012	Diabetes/Metabolism Research and Reviews	Clinical profiles and long-term outcomes of patients with pancreatic ductal adenocarcinoma and diabetes mellitus.	Conference abstract	Abstract, unable to find paper
31	LaVeccia, 1994	British J of Cancer	A case-control study of diabetes mellitus and cancer risk.	Wrong population	No real NOD, only < 5 y.
32	Hjalgrim, 1997	J of Internal Medicine	Cancer and diabetes—A follow-up study of two population-based cohorts of diabetic patients.	Wrong population	nothing about NOD; nothing about PC
33	He, 2017	Oncotarget	Serum metabolomics differentiating pancreatic cancer from new-onset diabetes.	Wrong outcomes	Biomarker study
34	Pan, 2018	American J of Epidemiology	Type 2 Diabetes and Risk of Incident Cancer in China: A Prospective Study among 0.5 Million Chinese Adults.	Wrong population	No PDAC group
35	Dakner, 2018	Diabetes/Metabolism Research and Reviews	Newly diagnosed type 2 diabetes may serve as a potential marker for pancreatic cancer.	Wrong population	No NOD
36	deJong, 2018	Cancer Epidemiology	Gastrointestinal cancer incidence in type 2 diabetes mellitus; results from a large population-based cohort study in the UK.	Wrong population	No NOD
37	Dong, 2018	Digestion	Predictive Factors for Differentiating Pancreatic Cancer-Associated Diabetes Mellitus from Common Type 2 Diabetes Mellitus for the Early Detection of Pancreatic Cancer.	Wrong population	Controls have long-standing diabetes.
38	Maitra, 2018	Pancreas	A Prospective Study to Establish a New-Onset Diabetes Cohort: From the Consortium for the Study of Chronic Pancreatitis, Diabetes, and Pancreatic Cancer.	Conference abstract/letter/review	This is a study protocol, not a study. The study is ongoing.
39	Ewald, 2012	Diabetes/Metabolism Research and Reviews	Prevalence of diabetes mellitus secondary to pancreatic diseases (type 3c).	Wrong population	
40	Fest, 2019	J of Internal Medicine	Erythrocyte sedimentation rate as an independent prognostic marker for mortality: a prospective population-based cohort study.	Wrong population	
41	Müller, 2018	Pancreas	The Potential of Glycemic Control and Body Weight Change as Early Markers for Pancreatic Cancer in Patients with Long-standing Diabetes Mellitus: A Case-Control Study.	Wrong population	
42	Eijgenraam, 2013	British J of Cancer	Diabetes type II, other medical conditions and pancreatic cancer risk: A prospective study in the Netherlands.	Wrong population	NOD is excluded.
43	LaTorre, 2014	BioMed Research International	Investigating the synergistic interaction of diabetes, tobacco smoking, alcohol consumption, and hypercholesterolemia on the risk of pancreatic cancer: A case-control study in Italy.	Wrong population	
44	Masclee, 2014	Gastroenterology	Comparison of incidence rates of acute pancreatitis and pancreatic cancer among the general population and type 2 diabetes mellitus patients between different databases in the safeguard project.	Conference abstract	Conference abstract Neither the first nor last author published anything about diabetes and cancer later.
45	Illés, 2014	Pancreatology	Benefits of screening for pancreatic cancer in new-onset diabetes mellitus.	Conference abstract	Conference abstract, follow-up publication is included
46	Freitas, 2014	Endocrine Reviews	Hospitalization and mortality for pancreatic cancer and diabetes: A cohort from a tertiary hospital.	Conference abstract	Conference abstract, publication was not found.
47	DeBruijn, 2014	Diabetologia	Diabetes and cancer risk in a population-based study with 20 years of follow-up: The Rotterdam Study.	Conference abstract/letter/review	Publication was by Fest, 2019
48	Ritchey, 2014	Pharmacoepidemiology and Drug Safety	Electronic health data capture of clinical evaluation and pancreatic cancer (PC) diagnosis (DX) in patients with type 2 diabetes (T2DM).	Conference abstract	Publication is under Brodovicz
49	Czakó, 2014	Pancreas	Screening for pancreatic cancer in new-onset diabetes mellitus is beneficial?	Conference abstract	Publication by Illes, 2016
50	Koo, 2019	Acta Diabetologica	Middle-aged men with type 2 diabetes as potential candidates for pancreatic cancer screening: A 10-year nationwide population-based cohort study.	Wrong population	No NOD
51	Larsson, 2005	Nature Publishing Group	Overall obesity, abdominal adiposity, diabetes, and cigarette smoking in relation to the risk of pancreatic cancer in two Swedish population-based cohorts.	Wrong population	No NOD
52	Fisher, 2001	World J of surgery	Diabetes: risk factor for developing pancreatic cancer or manifestation of the disease?	Review	Review of long-standing diabetes studies.
53	Chari, 2008	Gastroenterology	Pancreatic cancer-associated diabetes mellitus: prevalence and temporal association with diagnosis of cancer.	Wrong population	Their population is PDAC, not NOD
54	Makhoul, 2016	SAGE Open Medicine	Type 2 diabetes mellitus is associated with increased risk of pancreatic cancer: A veteran administration registry study.	Wrong population	NOD is excluded
55	Li, 2018	Medical Science Monitor: International Medical J of Experimental and Clinical Research	ABO Blood Group and Diabetes Mellitus Influence the Risk for Pancreatic Cancer in a Population from China.	Wrong population	No NOD
56	Müller, 2017	Pharmacoepidemiology and Drug Safety	HbA1c Levels, Body Weight Change, and Risk of Pancreatic Cancer Among Patients With Long-Standing Diabetes Mellitus: A Case-Control Study.	Conference abstract/letter/review	Conference abstract, study published by Müller, 2018
57	Khurana, 2004	American J of Gastroenterology	Diabetes Mellitus Is a Risk Factor for Pancreatic Cancer: A Case Control Study in Half a Million Veterans: 168.	Conference abstract/letter/review	Study published by Khurana, 2007. It is about medication.
58	Prizment, 2011	AACR	History of diabetes mellitus, cholecystectomy, and gallstone disease and risk of pancreatic cancer	Conference abstract/letter/review	Conference abstract, Study: Henry, 2012
59	Munigala, 2014	Gastrointestinal Endoscopy	1045 Higher Pancreatic Cancer Risk Following New Onset of Diabetes Mellitus in Non-Obese Patients with Chronic Pancreatitis.	Conference abstract	Conference abstract, paper is Munigala, 2015
60	Luo, 2007	Cancer Causes and Control	Body mass index, physical activity and the risk of pancreatic cancer in relation to smoking status and history of diabetes: A large-scale population-based cohort study in Japan—The JPHC study.	Wrong population	No NOD
61	Lo, 2013	International J of Cancer	Modest increase in risk of specific types of cancer types in type 2 diabetes mellitus patients.	Wrong population	No NOD
62	Luo, 2013	Cancer Causes and Control	Diabetes mellitus as a risk factor for gastrointestinal cancers among postmenopausal women.	Wrong population	No NOD
63	Lin, 2014	British J of Cancer	Independent and joint effect of type 2 diabetes and gastric and hepatobiliary diseases on the risk of pancreatic cancer risk: 10-year follow-up of population-based cohort.	Wrong population	No NOD
64	Liu, 2015	International J of Cancer	Cancer risk in patients with type 2 diabetes mellitus and their relatives.	Wrong outcomes	No other risk factors are studied
65	Christensen, 2016	Journal of Diabetes and its Complications	Venous thromboembolism and risk of cancer in patients with diabetes mellitus.	Wrong population	No NOD
66	Koo, 2019	J Clin Endocrinol Metab	The Incremental Risk of Pancreatic Cancer According to Fasting Glucose Levels: Nationwide Population-Based Cohort Study.	Wrong population	No NOD
67	Fritz, 2020	Int J Epidemiol	The triglyceride-glucose index as a measure of insulin resistance and risk of obesity-related cancers.	Wrong population	
68	Wlodarczyk, 2018	J Clin Gastroenterol	The Role of Insulin-like Growth Factor (IGF) Axis in Early Diagnosis of Pancreatic Adenocarcinoma (PDAC).	Conference abstract/letter/review	Review on IGF
69	Carey, 2013	Gastroenterology	The differential effects of statins on the risk of developing pancreatic cancer. A case-control study in two centers in the UK.	Wrong population	No NOD
70	Grote, 2011	Diabetologia	Diabetes mellitus, glycated hemoglobin and C-peptide levels in relation to pancreatic cancer risk: A study within the European Prospective Investigation into Cancer and Nutrition (EPIC) cohort	Wrong population	
71	Silverman, 1999	Nature Publishing Group	Diabetes mellitus, other medical conditions, and familial history of cancer as risk factors for pancreatic cancer	Wrong outcomes	
72	He, 2018	Current Medical Research and Opinion	Retrospective database analysis of cancer risk in patients with type 2 diabetes mellitus in China.	Wrong population	
73	Brodovicz, 2012	Diabetes, Obesity & Metabolism	Impact of diabetes duration and chronic pancreatitis on the association between type 2 diabetes and pancreatic cancer risk.	Wrong study design	
74	Johnson, 2011	Diabetologia	Time-varying incidence of cancer after the onset of type 2 diabetes: evidence of potential detection bias.	Wrong outcomes	No additional risk factors
75	Lee, 2019	Diabetes Care	Nationwide Trends in Pancreatitis and Pancreatic Cancer Risk Among Patients with Newly Diagnosed Type 2 Diabetes Receiving Dipeptidyl Peptidase-4 Inhibitors.	Wrong population	Population was patients taking DPP4 inhibitors
76	Fang, 2018	Endocrine Connections	Cancer risk in Chinese diabetes patients: A retrospective cohort study based on management data.	Wrong population	No NOD
77	Oberaigner, 2014	BMC Public Health	Increased cancer incidence risk in type 2 diabetes mellitus: Results from a cohort study in Tyrol/Austria.	Wrong outcomes	No additional risk factors
78	Antolino, 2022	European J of Surgical Oncology	Is TP53 Arg72Pro a risk factor for pancreatic cancer in diabetic patients?	Conference abstract	
79	Yuan, 2020	Diabetes	Is Type 2 Diabetes Causally Associated with Cancer Risk? Evidence From a Two-Sample Mendelian Randomization Study.	Wrong population	No NOD
80	Ma, 2022	J of Diabetes	Diabetes duration and weight loss are associated with onset age and remote metastasis of pancreatic cancer in patients with diabetes mellitus.	Wrong population	
81	Roxana, 2019	J of Gastrointestinal and Liver Diseases	Modifiable and non-modifiable risk factors for pancreatic cancer.	Conference abstract	
82	Shinyoji, 2020	Japanese J of Clinical Oncology	Diverse transitions in diabetes status during the clinical course of patients with resectable pancreatic cancer.	Wrong study design	
83	Van de Pall-Franse, 2007	International J of Cancer	Less aggressive treatment and worse overall survival in cancer patients with diabetes: a large population-based analysis.	Wrong population	
84	Everhart, 1995	JAMA	Diabetes mellitus as a risk factor for pancreatic cancer. A meta-analysis.	No original study	
85	Huxley, 2005	British J of Cancer	Type-II diabetes and pancreatic cancer: a meta-analysis of 36 studies.	No original study	
86	Pannala, 2008	Gastroenterology	Prevalence and clinical profile of pancreatic cancer-associated diabetes mellitus.	Wrong population	
87	Wang, 2006	Cancer Epidemiology, Biomarkers and Prevention	Diabetes mellitus and pancreatic cancer in a population-based case-control study in the San Francisco Bay Area, California.	Wrong outcome	no risk factors on top of NOD
88	Hassan, 2007	American J of Gastroenterology	Risk factors for pancreatic cancer: Case-control study.	Wrong population	No NOD
89	Kalapothaki, 1993	Cancer Causes Control	Tobacco, ethanol, coffee, pancreatitis, diabetes mellitus, and cholelithiasis as risk factors for pancreatic carcinoma.	Wrong population	No NOD
90	Canto, 2002	Gastroenterology	Screening for pancreatic neoplasia in high-risk individuals: The Johns Hopkins Experience.	No full text	
91	Chow, 1995	J of National Cancer Institute	Risk of pancreatic cancer following diabetes mellitus: a nationwide cohort study in Sweden.	Wrong population	NOD excluded
92	Gullo, 1994	NEJM	Diabetes and the risk of pancreatic cancer. Italian Pancreatic Cancer Study Group.	Wrong outcome	No risk factors on top of NOD
93	Bonelli, 2003	Pancreas	Exocrine pancreatic cancer, cigarette smoking, and diabetes mellitus: A case-control study in northern Italy.	Wrong outcome	No risk factors on top of NOD
94	Kim, 2014	Pancreatology	Serum CA 19-9 as a screening test for pancreatic cancer in new-onset diabetic patients.	No full text	Probably conference abstract
95	Cui, 2012	Endocrine-Related Cancer	Diabetes and pancreatic cancer.	Wrong population	
96	Atchinson, 2011	International J of Cancer	Risk of cancer in a large cohort of U.S. veterans with diabetes.	Wrong population	No NOD
97	Norell, 1986	British J of Cancer	Diabetes, gallstone disease, and pancreatic cancer.	Wrong population	No NOD
98	Olson, 2016	Pancreas	Weight loss, diabetes, fatigue, and depression preceding pancreatic cancer.	Wrong outcomes	No risk factors on top of NOD
99	Stapley, 2012	British J of Cancer	The risk of pancreatic cancer in symptomatic patients in primary care: a large case-control study using electronic records.	Wrong population	No NOD
100	Mueller, 2018	Pancreas	The potential of glycemic control and body weight change as early markers for pancreatic cancer in patients with long-standing diabetes mellitus: A case-control study.	Wrong population	
101	Aggarwal, 2012	Pancreatology	New-onset diabetes in pancreatic cancer: a study in the primary care setting.	Wrong population	Seemed to be the same study as Chari 2013
102	Chen, 2011	Diabetes Care	Risk of malignant neoplasm of the pancreas in relation to diabetes: A population-based study in Taiwan.	Wrong population	No NOD
103	Rousseau, 2006	International J of Cancer	Diabetes mellitus and cancer risk in a population-based case-control study among men from Montreal, Canada.	Wrong population	No NOD
104	Bao, 2011	Biochim Biophys Acta	The complexities of obesity, diabetes, and the development and progression of pancreatic cancer.	Basic research	
105	Wu, 2020	JAMA	Association of Glycated Hemoglobin Levels with Risk of Pancreatic Cancer.	Wrong outcome	No risk factors on top of NOD
106	Setiawan, 2019	National Cancer Institute	Pancreatic Cancer Following Incident Diabetes in African Americans and Latinos: The Multiethnic Cohort.	Wrong outcome	We tried to include it twice but found no data we could analyze, and authors did not respond
107	Keum, 2018	Cancer Causes Control	Long-term patterns of fasting blood glucose levels and pancreatic cancer incidence	Wrong outcomes	No risk factors on top of NOD

Explanation: “Wrong population” usually means that the study population are not NOD patients (most of the time, all diabetes patients instead), and no subgroup with NOD patients are examined for additional risk factors. “Wrong outcomes” usually means that no further risk factors are examined within the group of NOD.
